# Beyond Technocracy: How Capital Disparities Shape Gerontechnology Expectations Across Age Cohorts

**DOI:** 10.1093/geroni/igaf122.3547

**Published:** 2025-12-31

**Authors:** Yuhan Gao, Yajun Song

**Affiliations:** Peking University, Beijing, Beijing, China (People’s Republic); East ChinaUniversity of Science and Technology, Shanghai, Shanghai, China (People’s Republic)

## Abstract

Gerontechnology holds transformative potential for aging-in-place. However, its prevailing designs often reflect biomedical and technocratic biases, overlooking critical disparities in older adults’ technological engagement that fundamentally reflect inequities in cultural capital (electronic-health literacy and self-rated health) and social capital (intergenerational support). Grounded in Bourdieu’s capital theory, this study investigates how these multidimensional factors differentially shape expectations toward health-focused gerontechnology across age cohorts. We analyzed data from 869 Chinese older adults aged 60-99, categorized into three cohorts (60-64, 65-79, and 80-99). Path analysis was conducted to assess how electronic-health literacy, intergenerational support, self-rated health and other socioeconomic characteristics relate to older people’s expectations of two health-focused gerontechnology types: safety-monitoring and health-management. Distinct patterns emerged. The young-old cohort (60–64 years) with higher e-health literacy and poorer health status demonstrated significantly stronger expectations for both gerontechnology, whereas the middle-old cohort (65–79 years) with health declines primarily anticipated safety-monitoring benefits. The oldest-old cohort (80–99 years) revealed divergent dynamics—informational support increased expectations for both gerontechnology, yet reduced instrumental support correlated with health-management interest. These findings underscore that gerontechnology expectations are not merely a function of individual capability but reflect systemic inequities in lifelong capital accumulation. By moving beyond individual-centric adoption models, we argue for structural interventions that address disparities in e-health literacy and social support systems.

